# Multifocal Nonmetastatic Radioactive Iodine Avidity on Whole Body Scan After Thyroidectomy for Thyroid Cancer

**DOI:** 10.1016/j.aace.2024.07.001

**Published:** 2024-07-14

**Authors:** Jim T.C. Chen, Kirun Baweja, Lurdes Tse-Agha, Sara Awad

**Affiliations:** 1 Department of Medicine, Queen’s University, Kingston, Ontario, Canada; 2 Division of Endocrinology and Metabolism, Queen’s University, Kingston, Ontario, Canada

**Keywords:** differentiated thyroid carcinoma, thyroid cancer, whole-body scan, radioiodine, sodium-iodide symporter

## Abstract

**Background/Objective:**

Non-metastatic radioactive iodine (RAI) uptake can complicate the interpretation of whole-body scan (WBS) for differentiated thyroid carcinoma (DTC) post-thyroidectomy. We present a patient with DTC whose follow-up WBS showed nonmetastatic multifocal avidity in skeletal tissue, an uncommonly reported site of RAI uptake.

**Case report:**

A 42-year-old woman underwent a right hemithyroidectomy, followed by completion thyroidectomy and RAI remnant ablation therapy, for a 4.8 cm thyroid tumor consistent with stage pT3aNxMx follicular thyroid cancer. Follow-up WBS showed intense activity in the thyroid bed, right breast, left medial subcortical acetabulum, and several vertebral bodies. Her biochemical and clinical findings were not suggestive of cancer recurrence. Further workup with SPECT/CT and MRI showed no focal vertebral lesions and identified the left femoral lesion as a benign peripheral nerve sheath. Diagnostic mammography and ultrasound showed no evidence of suspicious breast lesions. Neck ultrasound was clear with no suspicious masses or pathologic lymphadenopathy. She remained in remission on continued active surveillance.

**Discussion:**

Nonmetastatic RAI uptake on WBS has many causes, including functional sodium-iodide symporter expression in nonthyroidal tissues, radioiodine accumulation in tissues and bodily fluids, and benign tumors. False-positive uptake can decrease the utility of post-treatment WBS in low-risk patients. Careful clinical examination, biochemical and radiologic follow-up, and close active surveillance can help distinguish false-positive uptake from metastatic or recurrent disease.

**Conclusion:**

We describe an uncommon case of RAI uptake in skeletal tissues after thyroidectomy for DTC, and we outline the steps taken to rule out underlying metastases.

**Highlights**

• While radioiodine whole-body scan (WBS) remains an important tool for the surveillance of patients with differentiated thyroid cancer, false-positive radioactive iodine (RAI) uptake can necessitate further biochemical and radiologic workup to rule out cancer recurrence or metastasis.
• False-positive RAI uptake can have a myriad of causes, including functional sodium-iodide symporter (NIS) expression, atypical NIS expression, tissue inflammation, bodily fluids, and benign tumors.
• SPECT/CT represents an important imaging modality following WBS as it provides RAI avidity data with anatomic localization to better characterize RAI-acid foci.

**Clinical Relevance**

We report an uncommon case of radioactive iodine (RAI) uptake in the skeletal tissues after thyroidectomy for differentiated thyroid cancer. We provide an introductory overview of the phenomenon of false-positive RAI uptake and detail the clinical, biochemical, and radiologic workup (including SPECT/CT) to rule out cancer recurrence and metastasis.

## Introduction

Radioactive iodine (RAI) iodine-131 (I-131) whole-body scan (WBS) is routinely used in the care of patients with differentiated thyroid carcinoma, as a method for treatment and post-thyroidectomy surveillance.^1^ This modality relies upon RAI uptake by thyroid tissues, which primarily occurs through sodium-iodide symporters (NIS). When RAI accumulates outside the expected physiologic distribution, the possibility of residual or metastatic disease must be ruled out. Nonmalignant foci on imaging can result from a variety of physiologic as well as pathophysiologic etiologies not directly related to thyroid cancer metastases or recurrence. False-positive, nonmetastatic RAI uptake must be differentiated from residual or recurrent thyroid cancer with a detailed history, physical exam, and investigations including biochemical markers and further localizing imaging if indicated. We present a case of a patient with false-positive nonmetastatic RAI uptake in skeletal tissues on WBS, which is rarely reported compared to other areas of false-positive uptake.

## Case Report

A 42-year-old woman was referred to the General Surgery clinic of an academic tertiary hospital by her primary care physician for assessment of multiple right-sided thyroid nodules with interval growth. Her past medical history includes bipolar disorder, stable on lamotrigine. She was not on any other prescription or over-the-counter medications.

Her symptoms started 10 years prior when she noted a right neck mass with mild discomfort. Her thyroid ultrasound at the time showed a single solid well-circumscribed right-sided nodule measuring 2.9 × 2.1 × 1.7 cm. As a result, she was referred to the General Surgery clinic and underwent fine needle aspiration biopsy of the single nodule, which showed benign follicular cells and inflammation with less than 1% risk of malignancy. She was subsequently discharged from the General Surgery clinic and closely monitored by her family physician with serial thyroid ultrasounds.

Eight years after initial symptom onset, repeat ultrasound showed a heterogeneously multinodular right hemithyroid without discrete borders; the largest nodule in the lower pole of the right hemithyroid measured 2.3 × 2.2 × 2.1 cm, with definite evidence of interval growth. Her left thyroid lobe measured 4.5 × 1.1 × 1.1 cm without clinically significant nodules, similar in size from 8 years prior.

At this point, she was re-referred to the General Surgery clinic. On clinical assessment, she denied dysphagia, dyspnea, or hoarseness of voice. She had no family history of medullary thyroid cancer, familial papillary thyroid cancer, or multiple endocrine neoplasia syndromes. There was no history of whole-body ionizing radiation exposure or nuclear accident exposure. Her workup showed a normal thyroid-stimulating hormone (TSH) of 0.88 mIU/L (normal 0.4-4.0 mIU/L). Physical exam of the thyroid noted right-sided thyroid fullness with a soft, irregular, and nodular texture. There was no lymphadenopathy noted. In the context of the increasing size of her right hemithyroid and increased discomfort, she opted for right hemithyroidectomy on an elective basis.

Her post-surgical pathology showed minimally invasive unifocal follicular carcinoma measuring 4.8 cm with clear surgical margins and without extrathyroidal extension, angioinvasion, or lymphatic invasion, stage pT3aNxMx, and stage I disease (based on the eighth edition of the American Joint Committee on Cancer TNM staging system^2^). One month after right hemithyroidectomy, she remained well with no constitutional symptoms or symptoms of thyroid dysfunction. Postoperatively, she was started on oral levothyroxine 25 mcg once daily due to a mildly elevated TSH of 4.8 mIU/L. She was subsequently referred to the Endocrinology Thyroid Cancer clinic by her General Surgeon for further management. On Endocrinology assessment, she was classified as low risk of recurrence given her pathology results and TNM stage. However, given the size of 4.8 cm, completion thyroidectomy was recommended to facilitate remnant ablation and long-term surveillance.

The patient underwent completion thyroidectomy 6 months later; the left thyroid was normal on pathology with no evidence of malignancy. She felt well postoperatively and was started on oral levothyroxine 150 mcg daily to maintain TSH at target (0.5–2.0 mIU/L) given her thyroid cancer risk category.^3^ Two months post-thyroidectomy, she underwent outpatient I-131 RAI remnant ablation therapy with 1125 MBq (30 mCi) of I-131 administered orally under thyrogen stimulation (0.9 mg administered intramuscularly 48 h and 24 h prior to RAI treatment, for a total of 2 doses of thyrogen).

**Fig. 1:**
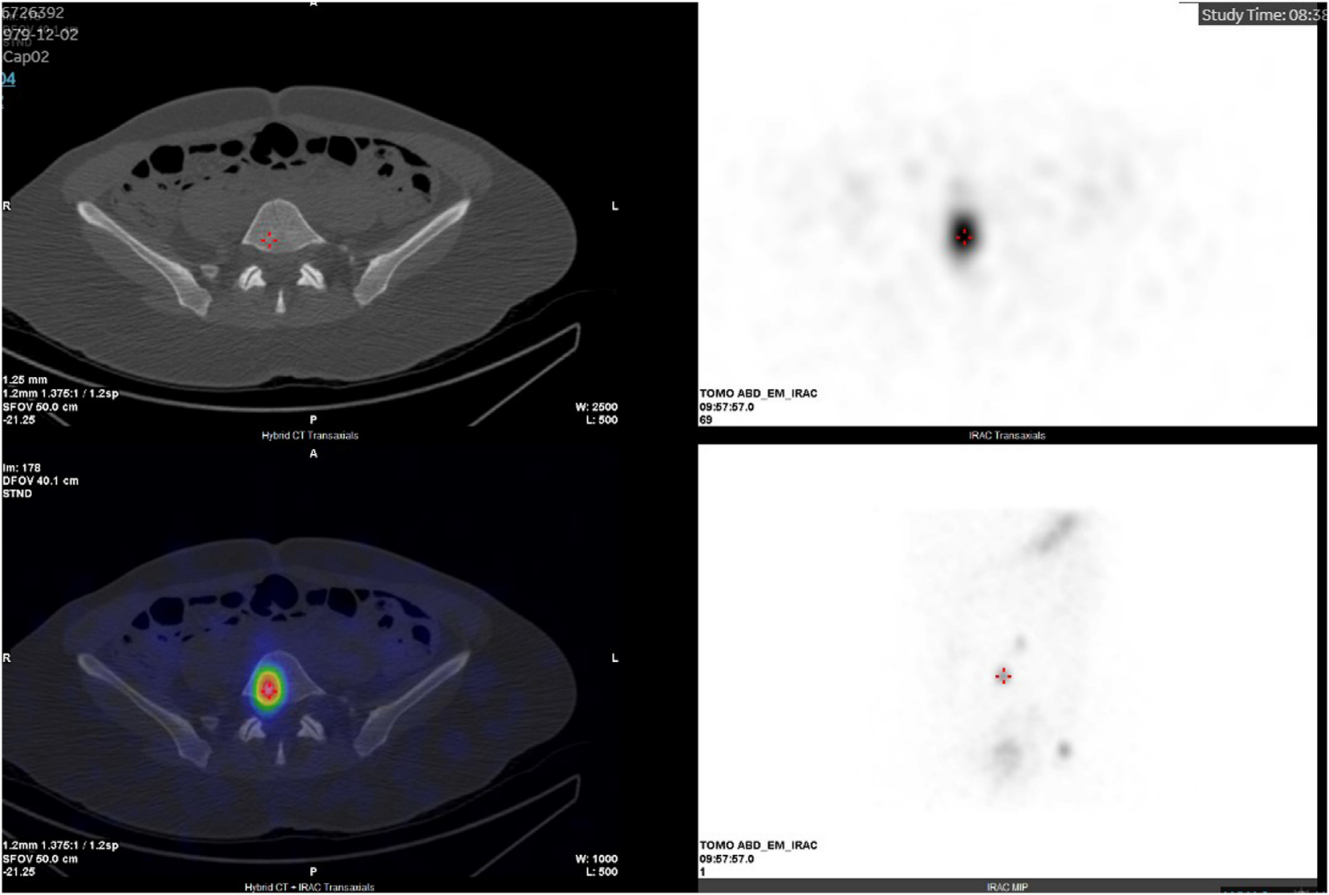
SPECT/CT and WBS (with overlap) showing nonmetastatic radioactive iodine uptake in the L5 vertebra. *WBS*, whole-body scan.

**Fig. 2:**
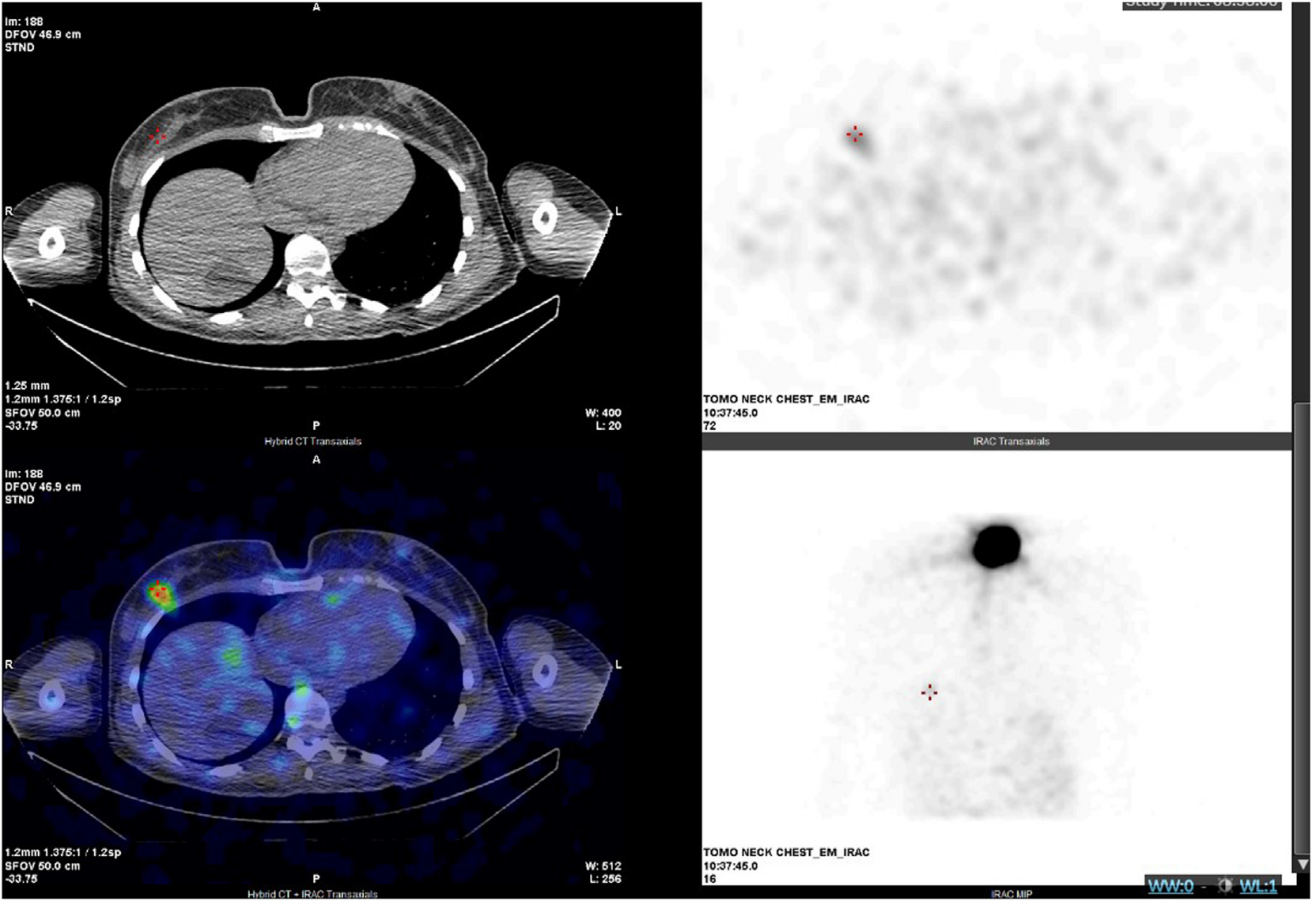
SPECT/CT and WBS (with overlap) showing nonmetastatic radioactive iodine uptake in the *right* breast. *WBS* = whole-body scan.

Her WBS 3 days after RAI ablation therapy showed intense activity in the thyroid bed (consistent with remnant thyroid tissue^4^) as well as multifocal I-131 avidity in several vertebral bodies (left seventh thoracic, left fourth lumbar, and right fifth lumbar) (Fig. 1), the medial subcortical acetabulum, and the right breast (Fig. 2). This prompted concern for possible metastatic disease versus physiologic uptake.

Although repeat bloodwork was requested 3 days post-I-131, the patient had forgotten and only performed bloodwork 17 days post-RAI ablation; her TSH was 0.25 mIU/Lwith negative anti-thyroglobulin antibodies and a thyroglobulin level of 8.6 μg/L (Table). Her neck and thyroid bed ultrasound were negative for residual disease or suspicious cervical lymphadenopathy.

**Table:** Biochemical Profile After Thyroidectomy and Radioactive Iodine (RAI) Remnant Ablation Therapy

*DATE*	TSH (0.40-4.50 mIU/L)	Thyroxine, free (9-22 pmol/L)	Thyroglobulin (3.5-77 μg/L)	Anti-thyroglobulin (<4 IU/mL)
Sept 27, 2021 (2 mo after completion thyroidectomy)	0.21	18	<0.1	<3
Nov 5, 2021 (pre-RAI administration)	77.2	20	1.6	<3
Nov 22, 2021	0.25	17	8.6	<3
Jan 6, 2022	0.36	17	<0.1	<3
Aug 22, 2022	0.52	17	<0.1	<4

Abbreviations: RAI = radioactive iodine; TSH = thyroid-stimulating hormone.

**Fig. 3:**
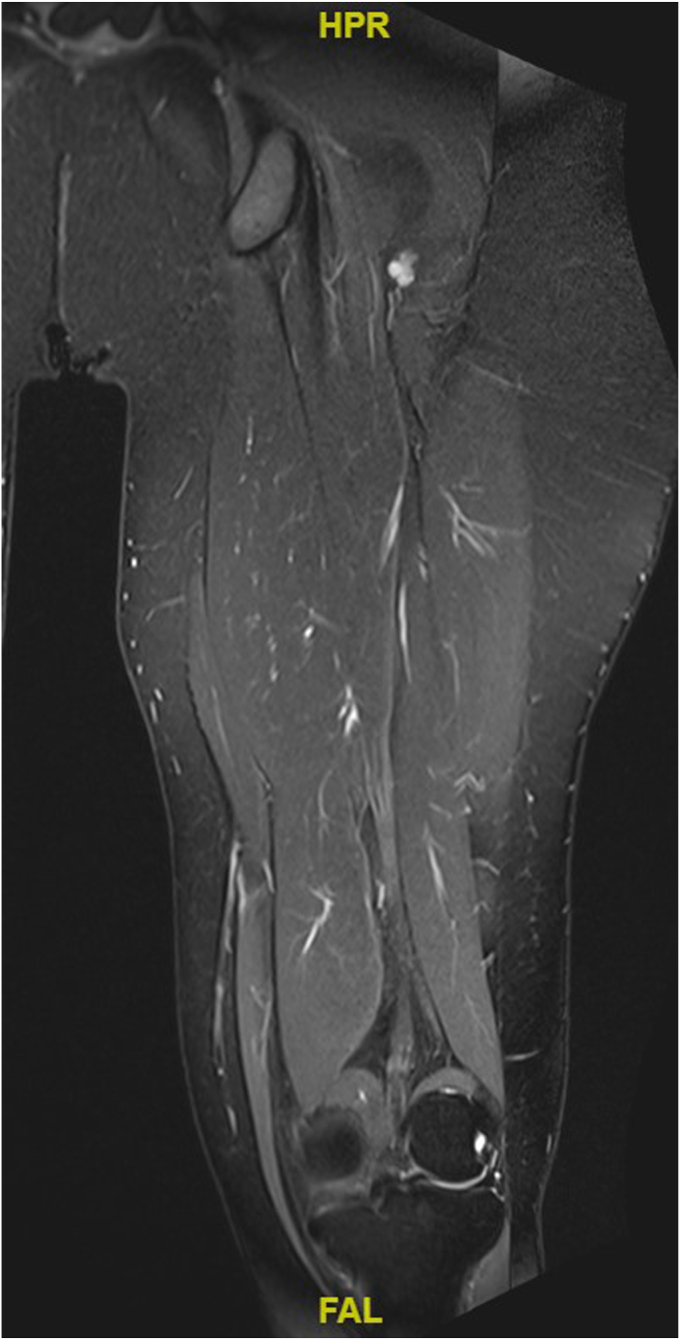
T2 MRI of the *left* thigh, showing a hyperintense lesion posterior to the proximal femur most consistent with a peripheral nerve sheath tumor. *MRI* = magnetic resonance imaging.

Further imaging studies were performed to characterize the foci of RAI uptake seen on WBS. Single-photon emission computed tomography/computed tomography (SPECT/CT) of the chest, abdomen, and spine revealed no corresponding morphologic abnormalities (Fig. 1 and 2). Magnetic resonance imaging (MRI) of the spine showed multilevel degenerative disc disease with no focal vertebral lesions involving the bone marrow. MRI of the pelvis showed a 11 × 5 × 16 mm lesion posterior to the left femur that remained stable in size over 3 months on repeat imaging, most consistent with a benign peripheral nerve sheath tumor (Fig. 3). Diagnostic mammography and breast ultrasound showed no suspicious breast lesions.

In the absence of morphologic abnormalities on correlating localizing scans, and no clinical or biochemical features of residual disease or metastases, the areas of RAI uptake in the neck, hip, vertebral bodies, and right breast were felt to represent non-metastatic avidity. The patient remained in clinical and biochemical remission on continued active surveillance. Her weight remained stable, and she had no palpable cervical lymphadenopathy. Her thyroid ultrasound and biochemical tests were likewise reassuring, with a TSH of 0.52 mIU/L and negative thyroglobulin (Table 1). While there was a plan for a subsequent thyrogen-stimulated WBS in 6 months to further confirm that all areas of uptake were nonmetastatic, this ultimately did not occur as the patient moved out of country and declined further testing as she was feeling well.

## Discussion

Iodide uptake through the basolateral membrane of follicular thyroid cells is essential for the biosynthesis of thyroid hormones. Iodide uptake is facilitated by the NIS, which couples the iodide uptake against its electrochemical gradient with the sodium uptake down its electrochemical gradient.^5^ Despite the scarcity of extracellular iodide, the NIS is highly effective at transporting iodide due to the symporter’s high affinity for iodide upon binding to sodium.^5^ This potent affinity underlies the basis for the WBS as a tool for monitoring DTC following total thyroidectomy.

False-positive uptake of I-131 on WBS is a well-documented phenomenon, commonly occurring in extrathyroidal tissues with functional NIS expression such as salivary glands, thymus, gastric mucosa, intestinal mucosa, and the breast.^5^, ^6^, ^7^ Other etiologies of RAI uptake include bodily fluids such as saliva, urine, menstrual blood, and feces,^1^^,^^6^, ^7^, ^8^ metabolism of radioiodinated thyroglobulin in the liver,^1^ tissue inflammation leading to locally increased capillary permeability and radioiodine accumulation,^1^^,^^6^^,^^8^ benign tumors, as well as nonthyroid malignancies (most commonly breast and lung cancers).^1^ Furthermore, individual variances in NIS expression can result in atypical areas of false-positive RAI uptake.^7^ For these reasons, the routine performance of post-treatment whole-body scans is increasingly questioned when the pretest probability of metastasis (based on risk classification and thyroglobulin level) is low, due to a predominance of false-positive uptake over true positives.^9^

False-positive uptake in the vertebrae is infrequently documented. Possible etiologies include vertebral hemangiomas,^10^ localized inflammation, bronchogenic cysts,^11^ or simply individual variances in NIS expression at the vertebrae. In this case, detailed imaging with SPECT/CT followed by spine MRI did not reveal any focal vertebral lesions, confirming that it is nonmetastatic uptake of I-131.

Biochemical testing with thyroid tumor markers (thyroglobulin and anti-thyroglobulin antibodies), as well as imaging with SPECT/CT, are helpful initial tools following WBS. SPECT/CT has become an increasingly common diagnostic tool for the postoperative management of thyroid cancer, and has been reported to change clinical management (ie, deciding whether to give RAI treatment, guiding extent of further surgical intervention, identifying need for further imaging) in significant numbers of patients.^4^ The clinical utility of SPECT/CT is attributed to the synergistic combination of radioiodine avidity data (from SPECT) and anatomic localization with attenuation correction (from CT), which makes it easier to differentiate between malignant and benign radioiodine uptake when compared to planar imaging modalities.^4^ The results from the SPECT/CT can also help guide further imaging (eg, ultrasound, MRI) if applicable to better characterize specific RAI avid foci.

Patient history and physical exam are crucial during postoperative management to assess for recurrence. In cases of thyroid cancer, signs and symptoms include cervical lymphadenopathy, new neck swelling, dysphagia, dysphonia, and constitutional symptoms.

In this case, the patient denied worrying symptoms during follow-up and her thyroglobulin level remained undetectable with negative anti-thyroglobulin antibodies and a negative neck and thyroid bed ultrasound. These were all reassuring for clinical, biochemical, and radiologic remission. Confirmatory localizing imaging (MRI of the spine and pelvis, diagnostic mammography, and breast ultrasound), biochemical testing, and continued active surveillance were successful at distinguishing the patient’s RAI foci as non-metastatic in nature, avoiding the need for more invasive diagnostic procedures.

## Conclusion

We reported an unusual case of avid RAI uptake in skeletal tissues that was ultimately identified as nonmetastatic avidity based on clinical context and further biochemical and imaging investigations, all of which were negative for metastatic disease. This report illustrates for clinicians the phenomenon of false-positive uptake on WBS post-RAI administration and next steps for imaging and biochemical testing. It also highlights that routine performance of post-treatment whole-body scans may be of questionable clinical value among patients with low risk disease.^9^ When radioiodine uptake is observed, a thorough history, physical examination, laboratory investigations, and additional imaging modalities can help clinicians distinguish false-positive uptake from recurrent or metastatic disease.

## Disclosure

The authors have no conflicts of interest to disclose.
